# Contrasting Community Composition of Active Microbial Eukaryotes in Melt Ponds and Sea Water of the Arctic Ocean Revealed by High Throughput Sequencing

**DOI:** 10.3389/fmicb.2020.01170

**Published:** 2020-06-03

**Authors:** Dapeng Xu, Hejun Kong, Eun-Jin Yang, Xinran Li, Nianzhi Jiao, Alan Warren, Ying Wang, Youngju Lee, Jinyoung Jung, Sung-Ho Kang

**Affiliations:** ^1^State Key Laboratory of Marine Environmental Science, Institute of Marine Microbes and Ecospheres, College of Ocean and Earth Sciences, Xiamen University, Xiamen, China; ^2^Fujian Key Laboratory of Marine Carbon Sequestration, Xiamen University, Xiamen, China; ^3^Division of Polar Ocean Science, Korea Polar Research Institute, Incheon, South Korea; ^4^Department of Life Sciences, Natural History Museum, London, United Kingdom

**Keywords:** assembly mechanism, community structure, diversity, high throughput cDNA sequencing, protist, 18S rRNA

## Abstract

Melt ponds (MPs), form as the result of thawing of snow and sea ice in the summer, have lower albedo than the sea ice and are thus partly responsible for the polar amplification of global warming. Knowing the community composition of MP organisms is key to understanding their roles in the biogeochemical cycles of nutrients and elements. However, the community composition of MP microbial eukaryotes has rarely been studied. In the present study, we assessed the microbial eukaryote biodiversity, community composition, and assembly processes in MPs and surface sea water (SW) using high throughput sequencing of 18S rRNA of size-fractionated samples. Alpha diversity estimates were lower in the MPs than SW across all size fractions. The community composition of MPs was significantly different from that of SW. The MP communities were dominated by members from Chrysophyceae, the ciliate classes Litostomatea and Spirotrichea, and the cercozoan groups Filosa-Thecofilosea. One open MP community was similar to SW communities, which was probably due to the advanced stage of development of the MP enabling the exchange of species between it and adjacent SW. High portions of shared species between MPs and SW may indicate the vigorous exchange of species between these two major types of environments in the Arctic Ocean. SW microbial eukaryote communities are mainly controlled by dispersal limitation whereas those of MP are mainly controlled by ecological drift.

## Introduction

One of the most characteristic features of the Arctic Ocean is its sea ice cover and annual cycling of freezing and melting of surface snow and sea ice. Melt ponds (MPs) are pools of open water that form on sea ice, glacial ice or ice shelves in the short Arctic summer. Areal coverage of MPs has been estimated to reach up to 80% of the Arctic sea ice in summer (Lüthje et al., 2006). Compared with sea ice/snow, MPs have a lower albedo so they absorb more heat, which constitutes one of the processes responsible for the polar amplification of global warming (Perovich et al., 2002; Flocco et al., 2012). MPs eventually disappear either by percolating through the sea-ice column or merging with sea water (SW) when the bottom of the pond reaches the ocean. They can also refreeze as the air temperatures drop again in the winter (Polashenski et al., 2012). Two different types of MP are usually found in the Arctic: open MPs which are connected with seawater and therefore show a high salinity (ca. 29), and closed MPs which comprise mostly freshwater and have a much lower salinity (Gradinger, 2002; Lee et al., 2012).

Melt ponds are estimated to contribute less than 5% to total annual production in the Arctic. Locally, however, they can contribute up to 30% of annual production, thus MPs are anticipated to play an important role in biogeochemical cycles (Fernández-Méndez et al., 2015). The formation, freezing, and merging with SW of the MPs can trigger the exchange of microbial eukaryotes among snow, sea ice, MP, and SW habitats in the Arctic Ocean (Hardge et al., 2017). Changes in the taxonomic and trophic structure of these communities can have a strong impact on key ecosystem functions, such as primary and secondary production, and element cycling. Studies on Arctic microbial eukaryotes, both from SW and sea ice, using conventional methods, e.g., light microscopy, flow cytometry, or high-performance liquid chromatography (HPLC), have been carried out for many years (Gradinger, 1999; Sherr et al., 2003; Niemi et al., 2011). However, the biodiversity of some species, e.g., pico/nano-sized or parasitic groups, which are small and lack sufficient morphological traits for accurate identification, is not well documented (Lovejoy, 2014). Nevertheless, recent studies have shown that the Arctic Ocean has active microbial food webs that are often dominated by cells <3 μm in size (López-García et al., 2001; Sherr et al., 2003), and that cells of <5 μm are responsible for much of the carbon fixation over wide regions of the Arctic Basin (Gosselin et al., 1997; Lee and Whitledge, 2005). Pico-sized cells have also been proposed to thrive as the Arctic Ocean freshens, this being one of the possible consequences of global warming (Li et al., 2009). More recently, culture-independent approaches, e.g., the sequencing techniques based on the extraction and amplification of environmental DNA, have enabled the acquisition of more complete picture of Arctic microbial eukaryotic communities from sea ice and SW (Eddie et al., 2010; Bachy et al., 2011; Piwosz et al., 2013; de Sousa et al., 2018). However, active microbial eukaryotes dwelling in MPs, and differences in their community assembly processes compared with SW, have been rarely studied, especially using culture independent methods such as high throughput sequencing (Kilias et al., 2014b; Hardge et al., 2017; de Sousa et al., 2018).

In the present study, we sequenced microbial eukaryotes based on total RNA extracts of samples from both MPs and SW in the Arctic Ocean. Using RNA instead of DNA extracts enabled us to target specifically the active assemblages, thus bypassing the bias from the dead/dormant cells, or extracellular DNA (Stoeck et al., 2007; Not et al., 2009; Orsi et al., 2013; Logares et al., 2014; Massana et al., 2015; Sun et al., 2016, 2017, 2019, 2020; Xu et al., 2017, 2018; Li et al., 2018). This study aimed to address the following questions: (1) do MPs and SW harbor distinct microbial eukaryotic communities and, if so, to what extent do they differ? (2) what are the major processes that control the assembly of microbial eukaryotic communities in MPs and SW?

## Materials and Methods

### Sample Collection and Measurement of Environmental Parameters

**FIGURE 1:**
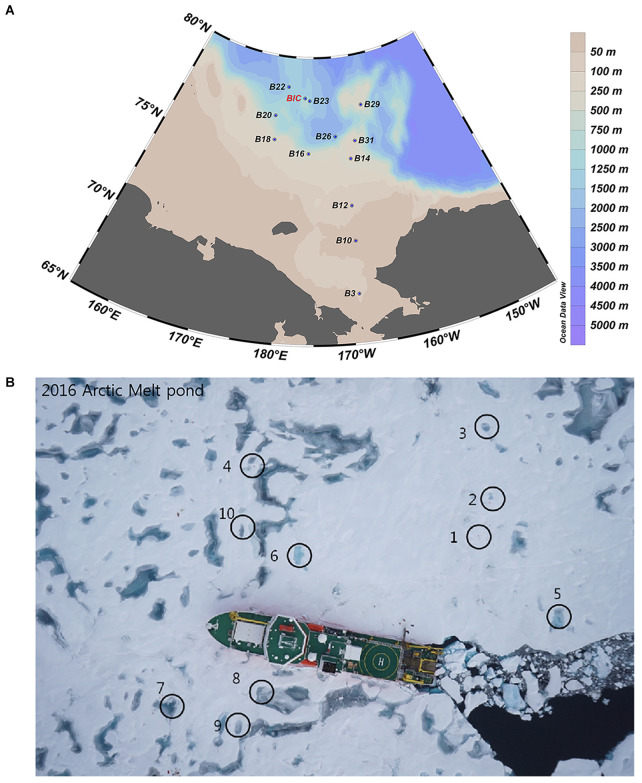
Sampling sites in the Arctic Ocean during the summer cruise of ARA07 conducted in 2016, showing the sea water **(A)** and melt pond **(B)** sites, respectively.

Samples were collected on board *IBRV ARAON* in Summer of 2016 (Expedition ARA07). A total of twelve SW sites and nine MPs (including two open, i.e., MP8 and MP9, and seven closed) were sampled (Figure 1). The cruise stations and sample identification numbers are included in Supplementary Table S1. Surface SW samples were collected using Niskin bottles which were set up in a circular rosette attached around sensors for measuring conductivity, temperature, and depth (Sea-Bird SBE 911plus, Sea-Bird Electronics, WA, United States). Surface water samples from the MPs were collected using polycarbonate bottles. Temperature and salinity were measured *in situ* using a water quality analyzer (YSI Pro2030, YSI Life Sciences, OH, United States).

Nutrients, i.e., nitrate + nitrite (NOx), phosphate (P), ammonium (NH_4_), and silicate (Si), were measured onboard using standard colorimetric methods adapted for use with a four-channel continuous auto-analyzer (QuAAtro; Seal Analytical, United States) according to the manufacturer’s instructions. Water samples (300–500 mL) were filtered through a cascade connection filtration system including 20-μm nylon mesh, a Nuclepore filter (Whatman International, United Kingdom) with a pore size of 2 μm, and a Whatman GF/F filter to collect the size-fractionated chlorophyll *a*, i.e., >20 μm, 2–20 μm, and <2 μm. Each filter was extracted in 90% acetone and chlorophyll *a* concentrations were measured with a fluorometer (Trilogy, Turner Designs, United States) previously calibrated against pure chlorophyll *a* (Sigma, United States).

Samples (2 ml) of 20 μm-mesh prefiltered seawater were fixed with 1% ice-cold glutaraldehyde and then deep frozen in liquid nitrogen. Pico-sized pigmented eukaryotes (PPE) were directly counted with a flow cytometer (Epics Altra II, Beckman Coulter, Brea, CA, United States). Heterotrophic bacteria (HB) were stained with SybrGreen I at 1/10,000 dilution and counted on the same flow cytometer following procedures described by Marie et al. (1999) and Jiao et al. (2014).

Water samples for nucleic acid extraction were prefiltered through 200 μm mesh Nitex (Sefar) to remove large metazoans. The micro- (20–200 μm), nano- (3–20 μm), and pico- (0.4–3 μm) sized fractions were sampled from ∼5 liters of water filtered sequentially with a peristaltic pump through 20 μm, 3 μm, and 0.4 μm pore size ISOPORE (Millipore) membrane filters, respectively, for less than 30 min to minimize RNA degradation. Filters were immediately frozen with liquid nitrogen and stored at −80°C for later RNA extraction. No replicates for nucleic acids extraction were used.

### RNA Extraction, PCR Amplification, and High Throughput Sequencing of the Hyper-Variable V4 Regions of the 18S rRNA

Total RNA was extracted from each cryopreserved filter membrane using RNeasy Mini Kit (Qiagen, United States) following the protocols of Sun et al. (2019). The RNA concentration and quality were determined using a Nanodrop spectrophotometer (Thermo Scientific, Wilmington, DE, United States) and gel electrophoresis, respectively. RNA was then reverse transcribed into cDNA using QuantiTect^®^ Reverse Transcription Kit and genomic DNA was removed by gDNA Wipeout Buffer supplied within the kit (Qiagen, China). The hyper-variable V4 region of the 18S rRNA (ca. 370 bp) was PCR amplified using cDNA as templates with primers TAReuk454FWD1 and TAReukREV3 (Stoeck et al., 2010). The PCR was run in four separate reactions for each sample to obtain sufficient amplicons for sequencing. The PCR conditions used were as follows: an initial incubation for 5 min at 94°C and then 30 cycles of 60 s at 94°C, 30 s at 55°C, and 30 s at 72°C, followed by a final extension step of 10 min at 72°C. The resulting PCR amplicons were excised from the gel and purified using MiniElute Gel Extraction Kit (Qiagen, United States). All purified amplicons were sent to Majorbio (Shanghai, China) for paired-end sequencing (2 × 250) using an Illumina MiSeq platform. Sequences obtained have been submitted to the NCBI Sequence Read Archive under the accession number PRJNA596339.

### Sequence Processing and Statistical Analysis

Quality filtering, demultiplexing and assembly of raw sequences were performed using Trimmomatic and Flash software (Magoć and Salzberg, 2011; Bolger et al., 2014) with criteria following Li et al. (2018). For each sample, quality-filtered reads were dereplicated using Usearch 11 (Edgar, 2010). Reads were denoised (i.e., reads with sequencing error were identified and corrected and chimeras were removed) and then clustered into biological zero-radius operational taxonomic units (ZOTUs) using UNOISE3 (Edgar, 2016b). ZOTUs that included fewer than four reads were removed from the dataset. The taxonomy assignment of ZOTUs was achieved using SINTAX (Edgar, 2016a) against the Protist Ribosomal Reference database (PR2, version 4.11.0, Guillou et al., 2012). Generation of ZOTU tables was done using *-otutab* command in USEARCH 11 following the removal of non-eukaryote-affiliated ZOTUs. Sequences were normalized for downstream analysis by randomly resampling at the lowest number of sequences recovered for all samples.

Alpha-diversity indexes, i.e., Richness, Shannon, Chao1, and Phylogenetic Diversity (PD), were calculated using QIIME (Caporaso et al., 2010). To infer differences between samples, Bray–Curtis distances were calculated for all samples and analyzed by Non-Metric Multidimensional Scaling (nMDS) in R using the “vegan” package. The Unweighted Unifrac metric was also used to infer the grouping of samples (Lozupone and Knight, 2005). The results were visualized using a two-dimensional Principal Coordinate Analysis (PCoA). Differences among groupings of samples were further tested by ANOSIM within PRIMER 6 (Clarke and Gorley, 2009). SIMPER (similarity percentage) analysis was used to identify ZOTUs primarily responsible for the differences observed among groupings of samples using Paleontological Statistics software (Hammer et al., 2001). The relationships between communities and environmental factors were explored with Mantel tests using the vegan package in R. Quantification of ecological processes, i.e., selection, dispersal and drift, were made according to the methodology described in Stegen et al. (2013), and Sun et al. (2019). Which first uses phylogenetic turnover between communities to determine the influence of selection, and then uses ZOTU turnover to determine the influences of dispersal and drift. First, phylogenetic turnover was measured by calculating the weighted ß-mean nearest taxon distance (ßMNTD), which indicate either communities are under heterogeneous selection or experiencing homogeneous selection. Null models were then constructed using 999 randomizations as in Stegen et al. (2013). Differences between the observed ßMNTD and the mean of the null distribution are denoted as ß-Nearest Taxon Index (ßNTI), which indicate either the deterministic processes or stochastic processes that drives the community assembly. Second, whether the observed ß diversity, based in OTU turnover, is generated by drift or other processes is determined by evaluating the Bray–Curtis based Raup-Crick metric for pairwise community comparisons by characterizing the magnitude of deviation between observed OTU composition turnover and null distribution of OUT composition turnover (Stegen et al., 2013).

## Results

### Environmental Parameters

**FIGURE 2:**
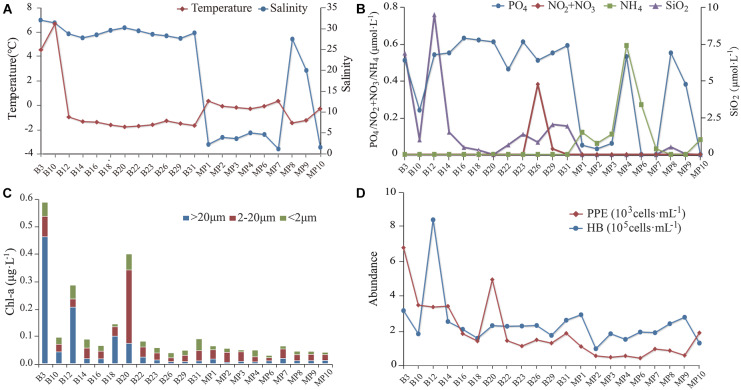
The environmental parameters measured at the sampling sites. **(A)** Water temperature and salinity. **(B)** Concentrations of NO_2_/NO_3_, PO_4_, NH_4_, and SiO_2_. **(C)** Size-fractionated chlorophyll *a* concentration. **(D)** Abundances of heterotrophic bacteria (HB) and pico-sized pigmented eukaryotes (PPEs).

Surface SW temperatures ranged from 6.5°C to −1.5°C. Water temperatures of the MPs ranged from −1.2 to 0.5°C (Figure 2A). Salinity of the SW ranged from 27.4 to 31.8. Salinity of the open MPs (MP8 and MP9) were 19.6 and 27.2, respectively, and those of the closed MPs ranged from 0.5 to 4.4 (Figure 2A). The concentrations of NO_2_/NO_3_ were below detection for all sites except B29. The concentrations of PO_4_ of the SW ranged from 0.25 to 0.63 μmol L^–1^ while those of the closed MPs ranged from 0 to 0.53 μmol L^–1^ and of the open MPs were 0.38 and 0.55 μmol L^–1^, respectively (Figure 2B). The concentrations of SiO_2_ of the SW ranged from 0.30 to 9.5 μmol L^–1^ while those of the closed MPs were below detection limit and of the two open MPs (MP8 and MP9) were 0.04 and 0.52 μmol L^–1^, respectively. The concentration of NH_4_ was below the detection limit in SW and open MPs and ranged from 0.03 to 0.59 μmol L^–1^ in the closed MPs.

The concentration of chlorophyll *a* (Chl *a*) was below 0.1 μg L^–1^ except at B3, B12, B18, and B20 (Figure 2C). In most sites, nano-sized plankton made the highest contribution to the total Chl *a* except at B3, B12, and B18 where the pico-sized plankton contributed the most. The abundance of HB at the SW sites was in the range 1.5–8.1 × 10^5^ cells mL^–1^ with the highest found at B12 while at the MP sites it was in the range 0.9–2.8 × 10^5^ cells mL^–1^. The abundance of PPE was about one order of magnitude lower than that of HB and was in the range 1.1–6.6 × 10^3^ cells mL^–1^ in the SW and 0.4–1.8 × 10^3^ cells mL^–1^ in the MPs (Figure 2D).

### Alpha Diversity of Microbial Eukaryotes

After quality screening and the removal of potential chimeras, reads that were not assigned as eukaryotes and ZOTUs that were represented by fewer than four reads, there were 4,011,421 reads remaining, ranging from 9,674 to 118,451 reads per sample (Supplementary Table S1). Rarefaction curves showed that for most samples there was not full recovery of microbial eukaryotes (Supplementary Figure S1). However, rarefaction curves for the pooled SW and the MP samples showed a symbol of saturation. After rarefied at a uniform sequencing depth based on the lowest sequence count (*n* = 9,674 sequences). A total of 1,697 ZOTUs was recovered from all samples, ranging from 25 to 493 ZOTUs.

**FIGURE 3:**
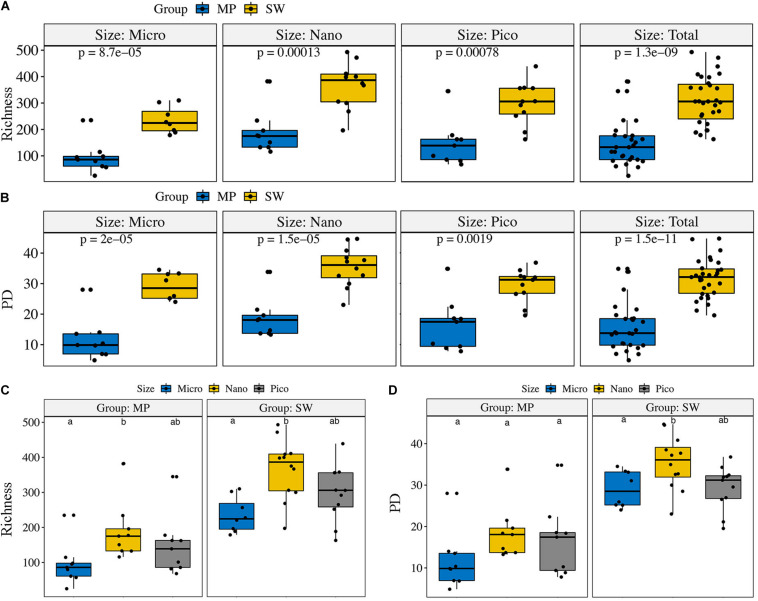
Alpha-diversity estimates (Richness and PD) for the size-fractionated **(A,B)** samples of the melt ponds (MPs) and sea water (SW) and for the pooled **(C,D)** MP and SW samples, respectively. The line in each box plot indicates the median, the box delimits the 25th and 75th percentile. Bars in **(C,D)** without shared letters indicate significant differences at the level of *p* = 0.05.

The ZOTU richness in the pooled and size-fractionated (micro-, nano-, and pico-) subcommunities was significantly lower in MPs than SW (Figure 3). The other three diversity indexes, i.e., Shannon, PD, and Chao1, showed the same trend (Figure 3 and Supplementary Figure S2). Within the size-fractionated MP and SW samples, nano-sized subcommunities usually have the highest diversity estimates, followed by the pico-, and micro-sized subcommunities (Figure 3 and Supplementary Figure S2).

### Beta Diversity and Community Composition of Microbial Eukaryotes

**FIGURE 4:**
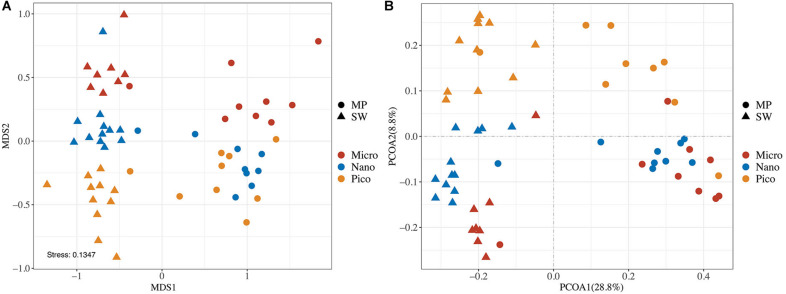
Plots of non-metric multidimensional scaling (nMDS) ordination based on Bray Curtis dissimilarities **(A)** and Unweighted UniFrac principal coordinates analysis (PCoA; **B**) of microbial eukaryotes based on community distance matrices.

**FIGURE 5:**
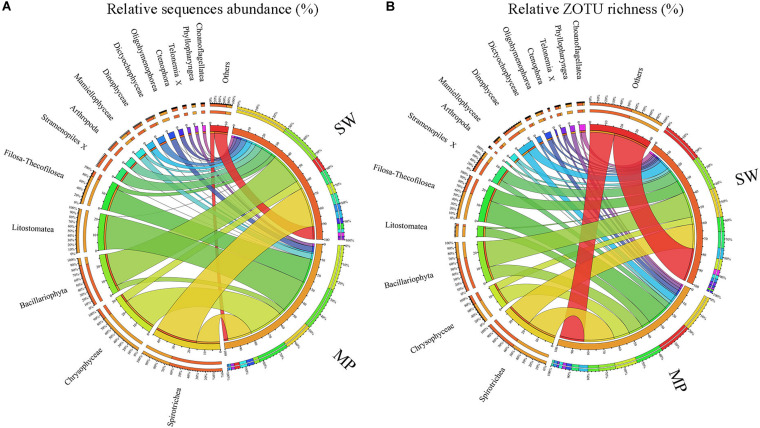
Overview of microbial eukaryotes in the pooled sea water (SW) and melt ponds (MPs). Relative sequence abuncance **(A)** and relative ZOTU richness **(B)**, respectively.

In the nMDS Ordination plot, all samples were clustered basically into two groups, the MP group and the SW group, the only exception being MP8, an open MP which grouped with SW (Figure 5A). This grouping was statistically supported (ANOSIM, *R* = 0.8160, and *p* < 0.0001). Within both the MP and SW groups, the subcommunities were basically separated by the size of the microbial eukaryote assemblages (Figure 4A). This clustering pattern was also supported by the two-dimensional PCoA plot of community taxonomic relatedness quantified by the Unweighted Unifrac metric (Figure 4B). Within both MP and SW groups, the size-fractionated subcommunities were statically significantly different from each other (Supplementary Table S2).

Overall, the MPs were characterized by having high relative sequence abundance of Chrysophyceae (ca. 25% of total MP sequences), the ciliate classes Litostomatea (ca. 24%) and Spirotrichea (ca. 16%), and the cercozoan groups Filosa-Thecofilosea (ca. 16%) with other groups contributing little to the total MP microbial eukaryote community. The SW was mainly dominated by Spirotrichea (ca. 29% of total SW sequences) and Bacillariophyta (ca. 23%), with other groups contributing less than 10% of total sequences individually (Figure 5A). In terms of ZOTU richness, ZOTUs affiliated with Spirotrichea contributed most (ca. 20% of total MP ZOTUs) to the MPs, followed by ZOTUs affiliated with Filosa-Thecofilosea (ca. 15%), Chrysophyceae (ca. 11%), and Bacillariophyta (ca. 9%). In the SW, the most abundant ZOTUs were members of Spirotrichea and Bacillariophyta, each of which contributed ca. 17% (Figure 5B).

**FIGURE 6:**
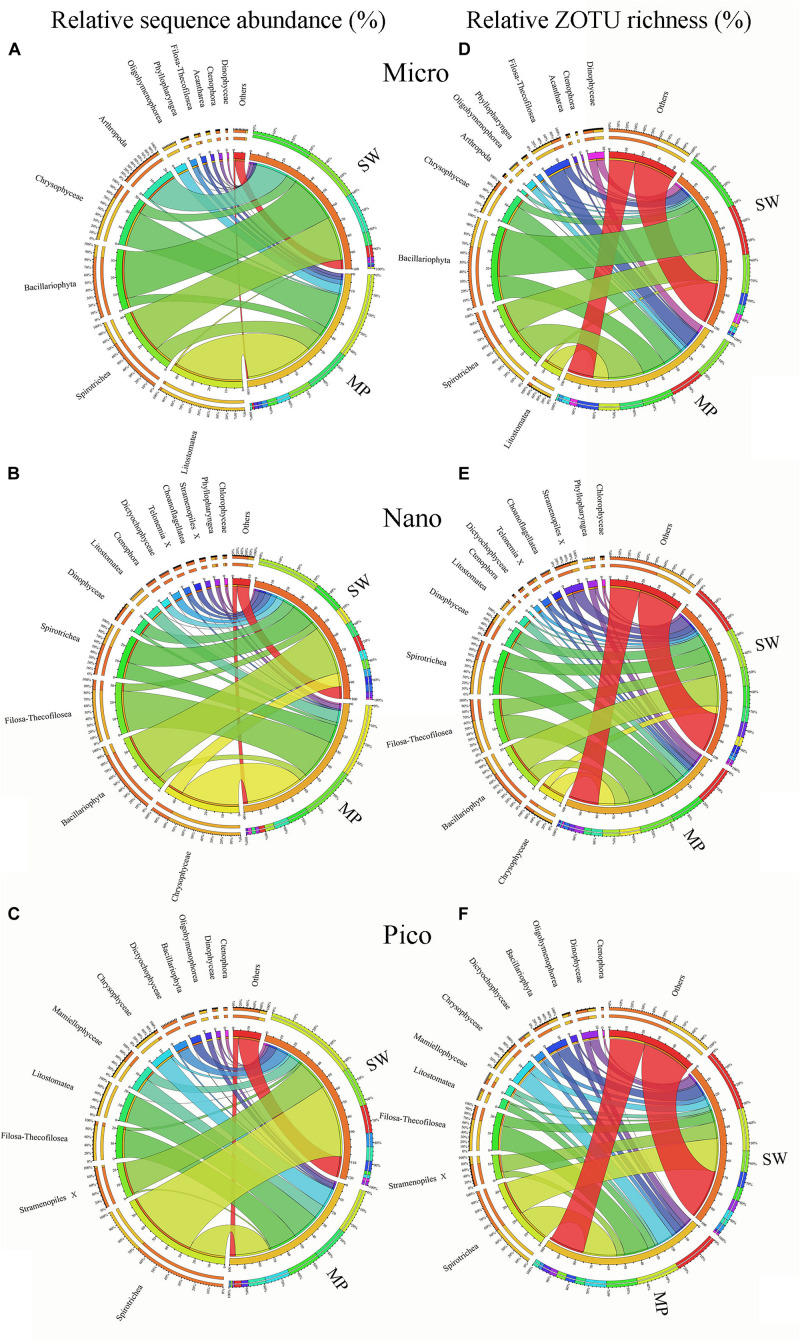
Overview of size-fractionated microbial eukaryotes in the sea water (SW) and melt ponds (MPs). Relative sequence abundance and ZOTU richness of micro- **(A,D)**, nano- **(B,E)**, and pico-sized **(C,F)** microbial eukaryote subcommunities.

Within the micro-sized fraction, the MP were characterized by high contributions of Litostomatea (ca. 44% of total reads), followed by Chrysophyceae (ca. 23%), and Spirotrichea (ca. 12%), with other lineages comprising the rest. The SW were characterized by high contributions of Bacillariophyta (ca. 31%) and Spirotrichea (ca. 29%), followed by Arthropoda (ca. 20%), and other lineages (Figure 6A). Within the nano-sized fraction of the MP, Chrysophyceae was the top contributor (ca. 38%), followed by Filosa-Thecofilosea (ca. 27%), Spirotrichea (ca. 11%), and other groups. Whereas in the SW, Bacillariophyta contributed the highest (ca. 33%), followed by Spirotrichea (ca. 16%; Figure 6B). Within the pico-sized fraction, Spirotrichea (ca. 26% of all reads) dominated the MP communities, followed by Litostomatea (ca. 20%), and Filosa-Thecofilosea (ca. 20%). Spirotrichea was the highest contributor (ca. 53%) to the SW communities followed by unidentified Stramenopiles (ca. 23%; Figure 6C). Large variabilities were found among the community composition of individual samples. For example, in the micro-sized community of the SW, the most dominant group was either Bacillariophyta or Spirotrichea, except B12 and B31 where Arthropoda was the dominant group (Supplementary Figure S3). In the nano-sized fraction of most MP samples, Spirotrichea was a minor component, although in MP4 it was the second most abundant group. Spirotrichea dominated most pico-sized SW samples, however, in B10 the most abundant group was Bacillariophyta.

In terms of ZOTU richness, Bacillariophyta and Spirotrichea dominated micro-sized SW communities, contributing almost half of all ZOTUs. In the MPs, the three most abundant groups were Spirotrichea, Chrysophyceae, and Litostomatea, which together constituted half of total ZOTUs (Figure 6D). The most abundant groups in the nano-sized subcommunities were Bacillariophyta (ca. 20%), Spirotrichea (ca. 10%), and Filosa-Thecofilosea (11%) in the SW, and Spirotrichea (ca. 18%), Filosa-Thecofilosea (ca. 17%), and Chrysophyceae (ca. 11%) in the MPs, respectively (Figure 6E). Spirotrichea and Stramenopiles accounted for ca. 25% and 10%, respectively, of all SW ZOTUs, being the top two contributors in the pico-sized subcommunities. In the MP pico-sized samples, Spirotrichea (ca. 22%) and Filosa-Thecofilosea (ca. 16%) were the two most abundant groups (Figure 6F). The ZOTU richness did not vary as much as sequence numbers in individual samples, but still showed some slight variation (Supplementary Figure S3).

The Venn diagram showed that 733 ZOTUs (43% of all ZOTUs) were shared between SW and MPs and ZOTUs exclusively found in SW and MPs were 796 and 168, respectively (Supplementary Figure S4A).

### Effects of Environmental Parameters on Community Structure of Microbial Eukaryotes

**FIGURE 7:**
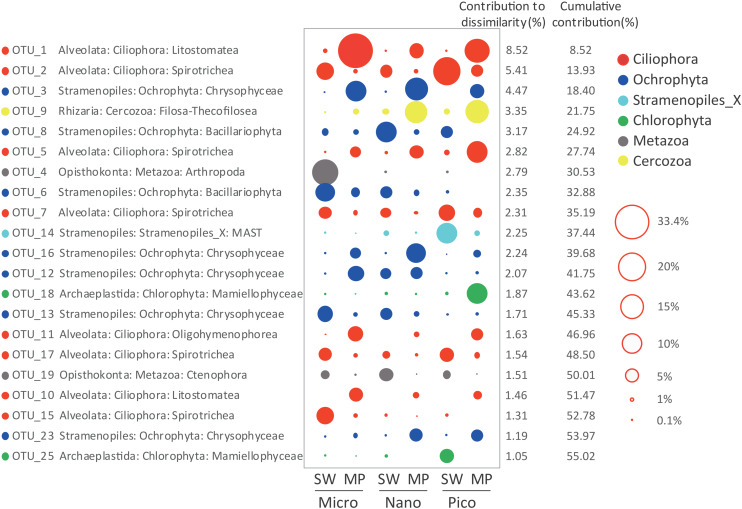
Taxonomic identities of the 21 ZOTUs that contributed most to community dissimilarities between sea water (SW) and melt ponds (MPs) with their relative contributions to community dissimilarities. The diameters of the circles are proportional to the abundances of the ZOTUs, with the size of the circle indicating the average abundance of each ZOTU at a given size of SW and MP groups.

SIMPER analysis selected 21 ZOTUs, which in total contributed ca. 55.02% of the dissimilarities in microbial eukaryote communities between the SW and MP groups. These ZOTUs were affiliated with Ciliophora (8 ZOTUs), Stramenopiles (8 ZOTUs), Metazoa (2 ZOTUs), Chlorophyta (2 ZOTUs), and Cercozoa (1 ZOTU), which contributed ∼24.5%, 19.45%, 4.3%, 2.92%, and 3.35% of the community dissimilarities, respectively (Figure 7). ZOTUs affiliated with Ciliophora were members of the classes Spirotrichea (4 ZOTUs), Litostomatea (2 ZOTUs), and Oligohymenophorea (1 ZOTU). ZOTUs identified as members in Stramenopiles were from Chrysophyceae (5 ZOTUs), Bacillariophyta (2 ZOTUs), and MAST (1 ZOTU). The ZOTUs identified as Cercozoa and Chlorophyta belonged to Filosa-Thecofilosea and Mamiellophyceae, respectively. The 2 ZOTUs identified as Metazoa were members of Arthropoda and Ctenophora, respectively (Figure 7).

The above 21 ZOTUs were also the most abundant ZOTUs in the pooled dataset, which in total contributed ca. 55.44% of total sequence counts (Table 1). To illuminate the ubiquity and identity of these ZOTUs, similarities were calculated between representative sequences of each ZOTU with its first BLAST hit (the nearest neighbor, NN), as well as the first BLAST hit with a species name (the nearest named neighbor, NNN) in GenBank. High similarities were found between representative sequences of ZOTUs and their NN that were all environmental sequences, 17 of which were identical and the rests had >99% similariy (Table 1). The locations where their NN was found were all oceanic sites with high latitudes, e.g., the Arctic Ocean, the Baltic Sea, and the Southern Ocean, the only exception being the NN of the most abundant ZOTU, ZOTU_1, which was found in mangrove waters of southern China. Eight ZOTUs were identical to their NNN and five had >99% similarity with their NNN. The lowest similarity was found between ZOTU_14, which was identified as a member of an environmental clade of MAST (Stramenopiles) and had 89.43% similarity with *Incisomonas marina* (GenBank accession number KY980417; Table 2).

**TABLE 1:** List of the most abundant 21 ZOTUs in the pooled dataset with the relative abundance of sequences, taxonomic identification, GenBank accession number of the nearest neighbor (NN), similarity (%-S) with NN, the location of where NN was reported, GenBank accession number and the identification of the nearest named neighbor (NNN), and similarity (%-S) with the NNN.

OTU ID	Relative abundance (%)	Group	GenBank accession no. of NN	%-S	Location of NN	GenBank accession no. of NNN	NNN	%-S
ZOTU_1	8.46	Ciliophora: Litostomatea	MH008816.1	100	Mangroves waters of Southern China	DQ487196.1	*Monodinium* sp. HCB-2005	99.03
ZOTU_2	6.05	Ciliophora: Spirotrichea	KJ762428.1	100	Arctic Ocean	KY980391.1	*Strombidium caudispina*	99.73
ZOTU_3	4.48	Stramenopiles: Chrysophyceae	EU545792.1	99.22	Surface layer sediments from the East Sea	JN934680.1	*Dinobryon faculiferum*	97.65
ZOTU_9	3.31	Cercozoa: Filosa-Thecofilosea	FN690368.1	100	The Baltic Sea	DQ303924.1	*Protaspis grandis*	98.71
ZOTU_8	3.16	Stramenopiles: Bacillariophyta	MK003433.1	100	Southern Ocean	KX253957.1	*Chaetoceros* cf. *socialis*	100
ZOTU_5	3.09	Ciliophora: Spirotrichea	FN689901.1	100	The Baltic Sea	KY980423.1	*Strombidium paracapitatum*	99.73
ZOTU_7	2.73	Ciliophora: Spirotrichea	MK003050.1	100	Southern Ocean	JX178818.1	*Pseudotontonia* sp. JG-2011a	100
ZOTU_4	2.43	Metazoa: Arthropoda	KJ761831.1	100	Arctic Ocean	MK921834.1	*Calanus glacialis*	100
ZOTU_6	2.36	Stramenopiles: Bacillariophyta	KJ761954.1	100	Arctic Ocean	MH843674.1	*Chaetoceros* sp.	100
ZOTU_14	2.17	Stramenopiles: MAST	HQ869375.1	100	Saanich Inlet	KY980417.1	*Incisomonas marina*	89.43
ZOTU_16	2.14	Stramenopiles: Chrysophyceae	JF698787.1	99.74	The Beaufort Sea	JN934680.1	*Dinobryon faculiferum*	96.87
ZOTU_12	2.09	Stramenopiles: Chrysophyceae	KT811095.1	100	Subsurface ocean observatory in Isfjorden	JN934680.1	*Dinobryon faculiferum*	100
ZOTU_17	1.84	Ciliophora: Spirotrichea	KJ762448.1	100	Arctic Ocean	KU525746.1	*Spirostrombidium apourceolare*	97.33
ZOTU_18	1.68	Chlorophyta: Mamiellophyceae	MF589928.1	99.47	The Kandalaksha Bay, the White Se	JF794053.1	*Mamiellaceae* sp. RCC2285	100
ZOTU_13	1.69	Stramenopiles: Chrysophyceae	EU545792.1	100	Surface layer sediments from the East Sea	JN934680.1	*Dinobryon faculiferum*	97.91
ZOTU_11	1.55	Ciliophora: Oligohymenophorea	FN689919.1	100	The Baltic Sea	HM030738.1	*Stokesia vernalis*	93.48
ZOTU_19	1.45	Metazoa: Ctenophora	HQ868938.1	100	Saanich Inlet	MF599320.1	*Ctenophora* sp.	100
ZOTU_10	1.39	Ciliophora: Litostomatea	FN689995.1	99.36	The Baltic Sea	MK056253.1	*Phialina caudata*	96.49
ZOTU_23	1.19	Stramenopiles: Chrysophyceae	MK003353.1	100	Southern Ocean	EF165133.1	*Ochromonas* sp. CCMP1899	99.74
ZOTU_15	1.18	Ciliophora: Spirotrichea	HM581790.1	100	Central Arctic Ocean	KY290321.1	*Ptychocylis minor*	99.46
ZOTU_25	1.00	Chlorophyta: Mamiellophyceae	MF589924.1	100	The Kandalaksha Bay, the White Sea	KY682863.1	*Micromonas polaris*	100

**TABLE 2:** Mantel test comparison between community variability (measured as Bray–Curtis dissimilarity) and environmental biotic and abiotic factors. When the correlation is significant both ρ-value and *R*^2^ are bold (*p* < 0.01).

Factor	Community distance	
	ρ	*R*^2^
Geographic distance	0.0001	0.338
Salinity	0.0001	0.809
Temperature	0.0001	0.558
PO_4_	0.0001	0.567
NO_2_+NO_3_	–0.050	0.841
NH_4_	0.0001	0.470
SiO_2_	0.0001	0.357
Chl *a*	0.0001	0.257
Heterotrophic bacteria	0.0001	0.227
Pigmented pico-sized eukaryotes	0.0001	0.316

The influence of environmental parameters on the microbial eukaryote communities was analyzed by the Mantel test. Salinity was identified to be the dominant driving factor (*p* < 0.0001, *R*^2^ = 0.809). The massive co-variance of biotic and abiotic factors with salinity enables them also to be driving factors.

### The Assembly of Microbial Eukaryotes Communities

To further assess the contributions of spatial and environmental factors on microbial eukaryote community structure, quantification of ecological processes mediating community assembly was performed. Dispersal limitation was found to be the primary driver for the community assembly processes of SW microbial eukaryotes and explained 71% of community turnover, followed by heterogeneous selection (ca. 17%), and drift (ca. 11%). In the MPs, drift contributed ca. 63% of microbial eukaryotic community turnover, followed by dispersal limitation (ca. 20%), selection (heterogeneous and homogeneous selection, ca. 9%), and homogenizing dispersal (ca. 7%).

## Discussion

### Environmental Parameters at the Sampling Sites

The temperature of the closed MPs (MP8 and MP9) was higher than the SW and that of the open MPs was similar to the SW. The salinity of the MPs was much lower than that of the SW, averaging 2.5 in the closed MPs and 23.4 in the open MPs. The temperature and salinity of the MPs were within the range of previous reports on both open and closed MPs (Gradinger et al., 2005; Lee et al., 2012; Lin et al., 2015; Hardge et al., 2017; Sørensen et al., 2017).

The concentrations of NO_3_/NO_2_ were below the detection limit for all stations except B26. The concentrations of PO_4_ and SiO_2_ were lower in the MPs than the SW which is consistent with previous studies (Lee et al., 2012; Sørensen et al., 2017). The concentrations of NH_4_ for all SW and open MPs were below the detection limit. For the closed MPs, NH_4_ concentrations were variable but always <1 μmol L^–1^ which is consistent with that reported previously (Lee et al., 2012). The Chl *a* concentrations of SW varied significantly among sites but were within the ranges of previous reports (Lee et al., 2010; Comeau et al., 2011; Lavrentyev et al., 2019). The Chl *a* concentrations of all MPs were <0.1 μg L^–1^, which is lower than previous studies (Lee et al., 2012; Gourdal et al., 2018). Community succession was probably the major factor that caused these differences in Chl *a* concentrations.

The abundances of PPE and HB at most SW sites were within the ranges of previous reports from the Arctic Ocean and were of the same magnitude as abundances from tropical/subtropical and boreal open oceans (Massana, 2011). The abundances of HB in MPs were within the ranges of previous HB counts in MPs (Gourdal et al., 2018).

### Alpha Diversity and Community Composition of Microbial Eukaryotes in Sea Water and Melt Ponds

Since the landmark work of López-García et al. (2001), surveys of microbial eukaryotes in polar regions have routinely used culture-independent, i.e., sequencing-based, methods. Consequently, studies applying rDNA analyses have been carried out to reveal the biodiversity of microbial eukaryotes from a variety of polar environments including SW, ice, snow, and sediments (Lovejoy et al., 2006; Tian et al., 2009; Bachy et al., 2011; Comeau et al., 2011; Kilias et al., 2013, 2014a; Monier et al., 2013; Jung et al., 2015; Stecher et al., 2016; Gast et al., 2018). Overall, the SW microbial eukaryotic communities in the present survey were dominated by Ciliophora and Bacillariophyta (Figure 5) but large variabilities were found among size-fractionated communities and individual samples (Figure 6 and Supplementary Figure S3). Previous studies have also shown huge spatial and temporal variations of SW microbial eukaryotic communities among different SW environments in the Arctic Ocean using either DNA-based or RNA-based (or both) sequencing (Comeau et al., 2011, 2019; Lovejoy and Potvin, 2011; Balzano et al., 2012; Monier et al., 2013; Zhang et al., 2019).

Few studies have been carried out to reveal the community composition of MP microbial eukaryotes (Kilias et al., 2014b; Hardge et al., 2017). In the present study, size-fractionated subcommunities as revealed by RNA-based HTS showed contrasting compositions between MPs and SW. Significantly lower alpha diversity estimates were found in the MPs than SW across all size fractions (Figure 3 and Supplementary Figure S2). Based on microscopy observations, it was found that phytoplankton diversity was significantly higher in the surface SW than the closed MPs (Lee et al., 2011). Our study is consistent with another previous study which showed that protist OTU richness was lower in MPs than in the deep chlorophyll maximum layer, ice, and under-ice water (Hardge et al., 2017). The same study found that there was high variability in community composition among individual MPs which is consistent with present findings (Supplementary Figure S3).

The present study showed that micro-sized active microbial eukaryotic communities were dominated by Ciliophora (represented mainly by Litostomatea, Spirotrichea, and Oligohymenophorea) and Chrysophyceae. The nano-sized fraction was dominated by Chrysophyceae and Filosa-Thecofilosea (Cercozoa), followed by Ciliophora. In the pico-sized community, Ciliophora, Cercozoa, Mamiellophyceae, and Chrysophyceae together contributed >90% of the reads. Using DNA-based sequencing, Hardge et al. (2017) found that Chrysophyceae (e.g., *Ochromonas*), Bacillariophyceae, and Ciliophora (e.g., *Didinium*, *Paramecium*) dominated MP communities, which is consistent with present findings. Another study showed that protist communities in MP aggregates were dominated mainly by Chlamydomonadales, Chrysophytes, and Dinoflagellates, and cell counting by flow cytometry showed that most of these cells were within the size range 3–10 μm (Kilias et al., 2014b). The same study also reported that OTUs classified as *Dinobryon faculiferum* were only abundant in MP aggregates but not in the sea ice bottom layer. In our study, several ZOTUs having the closest named match (NNN) as *D. faculiferum* were also found to be abundant in the MPs (e.g., ZOTU_3, 12, 13, and 16, Table 1). Most of these ZOTUs were recovered from the nano/pico-sized fractions of the MP communities, except ZOTU_13, which was more prominent in micro-sized fraction of SW (Figure 7). Our data could serve as evidence that the aggregates were probably formed by physical aggregation processes in the MPs in stead of the sea ice (Kilias et al., 2014b).

A previous study using both RNA- and DNA-based pyrosequencing on sea ice protist communities found higher representation of Ciliophora (Stecher et al., 2016). This may have been due to a high potential metabolic activity of ciliates in the sea ice and/or the high copy number of 18S rRNA gene of ciliates (Gong et al., 2013; Surprisingly, ciliate-affiliated sequences, mostly representing Litostomatea (e.g., *Didinium*) and Spirotrichea, were abundant not only in the micro-sized but also in the pico-sized MP communities (Supplementary Figure S3). A total of 293 ZOTUs belonging to Ciliophora were found in MPs, among which 87 (ca. 29.7%) were shared by all three size fractions and 43 (ca. 14.7%) were found exclusively in the pico-sized fraction (Supplementary Figure S4B). To the best of our knowledge, no pico-sized ciliates have ever been identified and described using microscopy-based approaches. This could be due to flexible cells that squeezed through the 3-μm filter pores and/or to cell breakage during sample collection. However, we cannot rule out the possibility that pico-sized ciliates do exist in the Arctic Ocean considering the fact that: (1) ciliates (mainly naked oligotrichs) as small as 12–15 μm have been found in the Arctic Ocean (Lynn et al., 1991; Sherr et al., 2013); (2) small ciliates (ca. 20 μm) are reported to be widely distributed and occasionally dominate microzooplankton communities in oligotrophic oceans (Pierce and Turner, 1992); and (3) the lack of rigorous morphological surveys of ciliates in the polar regions compared with other ocean regimes (Petz et al., 1995). Overall, the high relative sequence abundance of raptorial ciliates, such as *Didinium-*affiliated ZOTUs, in MPs is probably due to the absence of marine metazoans, one of the major top down control factors of ciliates in marine ecosystem, owing to the near-freshwater conditions (Kramber and Kiko, 2011; Lee et al., 2015).

Eighteen out of the 21 most abundant ZOTUs found in our dataset are identical to their NN in GenBank which indicates that they were probably found in other marine samples and not restricted to the area sampled here. Also, the locations where their NN were found were all high latitude ocean sites indicating their wide distribution in cold oceanic waters. The only exception was ZOTU_1, the NN of which was found in mangrove waters of the South China Sea. ZOTU_1 has 99.03% similarity with its NNN, i.e., *Monodinium*, species of which are also found in polar and other aquatic environments (Hada, 1970; Foissner et al., 1999; Hardge et al., 2017). Among the 21 ZOTUs, 8 have relatively lower (<99%) similarities with their NNN which indicates a large undiscovered/undescribed diversity of microbial eukaryotes in the Arctic Ocean (Table 1).

### The Exchange of Microbial Eukaryotes Between Melt Pond and Sea Water

The annual cycle of freezing and melting of SW causes large variations of physical and biological properties of the SW and the sea ice that will lead to shifts in community composition and exchange of freshwater and marine organisms (Li et al., 2009; Tremblay et al., 2009; Kilias et al., 2014b; Hardge et al., 2017). A previous study analyzing protist communities within sea ice, MPs, under-ice water and deep-chlorophyll maximum water at a number of sea ices stations showed low exchange among the four habitats during sea ice melting, but high exchange during new sea ice formation (Hardge et al., 2017). In the latter case, protists dwelling in MPs contributed most significantly to the overall exchange (Hardge et al., 2017). Our study, which employed RNA-based rather than DNA-based sequencing, showed that ca. 38.7% of all ZOTUs were shared between SW and open MPs, and ca. 25.1% were shared between SW and closed MPs (Supplementary Figure S4C). These findings are consistent with those of Hardge et al. (2017) who reported 26% of all OTUs (recovered by DNA-based sequencing) were shared between MPs and under sea ice water. One MP, i.e., MP8, which had similar physical/chemical properties to SW, grouped with SW rather than MP samples. MP8 was an open MP which could be at an advanced stage of development. Consequently, the microbial eukaryotic community was more influenced by the adjacent SW which will eventually merge with the ocean. Our study, although lacking data for SW and MP microbial eukaryotic communities during new sea ice formation, could be used to inform future studies on the impact of sea ice/snow melting on overall community dynamics in the Arctic Ocean.

### Assembly of Microbial Eukaryote Communities in Sea Water and Melt Ponds

**FIGURE 8:**
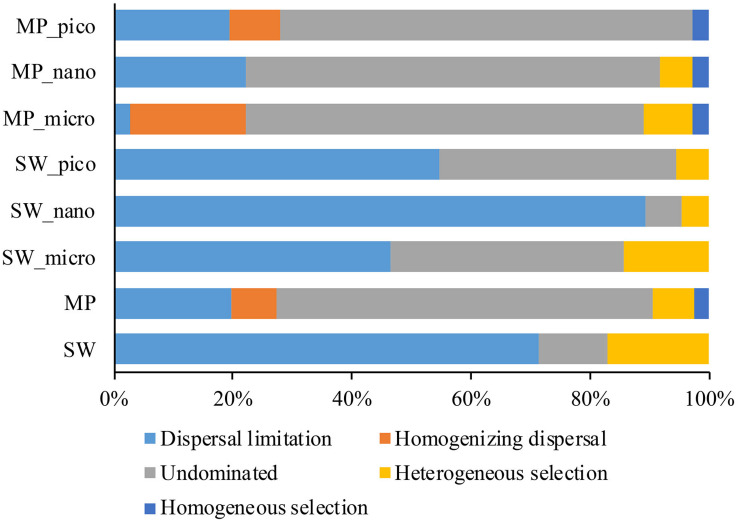
Partition of community assembly process of microbial eukaryotes in the size-fractionated, and pooled sea water (SW), and melt pond (MP) samples.

Previous studies have mainly used DNA-based sequencing to infer community assembly mechanisms of marine microbial eukaryotes (Wu et al., 2017; Logares et al., 2018; Wu and Huang, 2019). Consequently, the findings of such studies may have been influenced by DNA from dormant/dead microbial eukaryotes and extracellular DNA. In order to mitigate this problem, the present study employed RNA-based HTS to reveal the community assembly mechanisms of microbial eukaryotes in the Arctic Ocean. Contrasting assembly mechanisms for MP and SW microbial eukaryote communities were revealed. The SW microbial eukaryote communities were predominantly structured by dispersal limitation (ca. 71.4% of the turnover) whereas the MP communities were shaped mainly by drift (ca. 63.3% of the turnover; Figure 8). These findings are consistent with those of Wu et al. (2017) who, based on samples collected from the East and South China Sea, found that the picoeukaryotic communities of the surface ocean were primarily influenced by dispersal limitation. The SW stations sampled in the present study were located in the Chukchi Sea. Although the waters in the Arctic Ocean are connected, the different sources of water, e.g., the cold, relatively fresh water arriving from the Pacific Ocean through the Bering Strait, freshwater runoffs from adjacent land, meltwater from glaciers and sea ice, and waters from the north Atlantic Ocean, may serve as a barrier and limit dispersal of protists (Jones, 2001; Rudels, 2015). For the MPs, drift was identified to be the dominant process determining the structure of microbial eukaryote communities (∼63.3% of the turnover), which was ca. 3.2 times that of dispersal limitation (Figure 8). These findings are similar to those of Logares et al. (2018) who reported that the microbial eukaryote communities were predominantly structured by drift (ca. 72% of the turnover), which was ca. 3 times more important than dispersal limitation. This latter study was carried out on planktonic microbial eukaryotes in lakes in Eastern Antarctica, which emerged from the sea during the last 6000 years. Although the salinity ranged from freshwater to hypersaline (salinity 250) in the studied lakes, the effects of salinity along with other environmental variables on microbial eukaryote community structure were not significant, indicating a minor role of selection on the assembly of lacustrine microbial eukaryote communities (Logares et al., 2018). The melting of snow during the short Arctic summer leads to the formation of MPs on the sea ice, which generally are not connected to the under-ice water (Sankelo et al., 2010). As the MPs develop, some will melt through the sea ice below, connect with the under-ice water and become open MPs. The MPs eventually either disappear, either by percolating through the whole sea-ice column, merging with SW when the bottom of the pond reaches the ocean, or refreezing as the air temperatures drop again in the winter (Polashenski et al., 2012). As the snow/sea ice melts, microbial eukaryotes in the snow and sea ice are released into the MPs (Hardge et al., 2017). During the freezing of MP water and sea ice formation, microbial eukaryotes in the MPs can be passively trapped within the sea ice matrix (Arrigo et al., 2010). Ecological drift is associated to stochastic changes in the relative abundance of taxa. In the present study, the microbial eukaryotic assemblages in the MPs showed higher similarity of community composition than those of the SW (Supplementary Figure S5) which may partially be explained by the stochastic process (ecological drift). However, selection has also been found to structure the MP microbial eukaryote communities (ca. 9.4% of total turnover). It is noteworthy that the number and geographic area of the MPs sampled in the present study were limited and further studies including more MPs from larger geographic areas are needed to validate the current findings.

## Data Availability Statement

This article contains previously unpublished data. The sequence data have been submitted to the NCBI Sequence Read Archive under the accession number PRJNA596339.

## Author Contributions

DX and E-JY conceived and designed the study. E-JY collected the samples. HK and XL conducted the experiments. DX, HK, and YW analyzed the data. YL, JJ, and S-HK performed the nutrients and flow cytometry analysis. All authors wrote the manuscript.

## Conflict of Interest

The authors declare that the research was conducted in the absence of any commercial or financial relationships that could be construed as a potential conflict of interest.
