# Supplementary material for: Contrasting Community Composition of Active Microbial Eukaryotes in Melt Ponds and Sea Water of the Arctic Ocean Revealed by High Throughput Sequencing
Source: Front Microbiol. 2020 Jun 3;11:1170. doi: 10.3389/fmicb.2020.01170 (PMC7291953; doi:10.3389/fmicb.2020.01170)
Supplement: Supplementary file 1 [file Data_Sheet_1.docx]

**FIGURE S1.** Rarefaction analysis of microbial eukaryote 18S rRNA gene sequences in individual (**A**) and pooled sea water (SW) and melt pond (MP) samples (**B**).


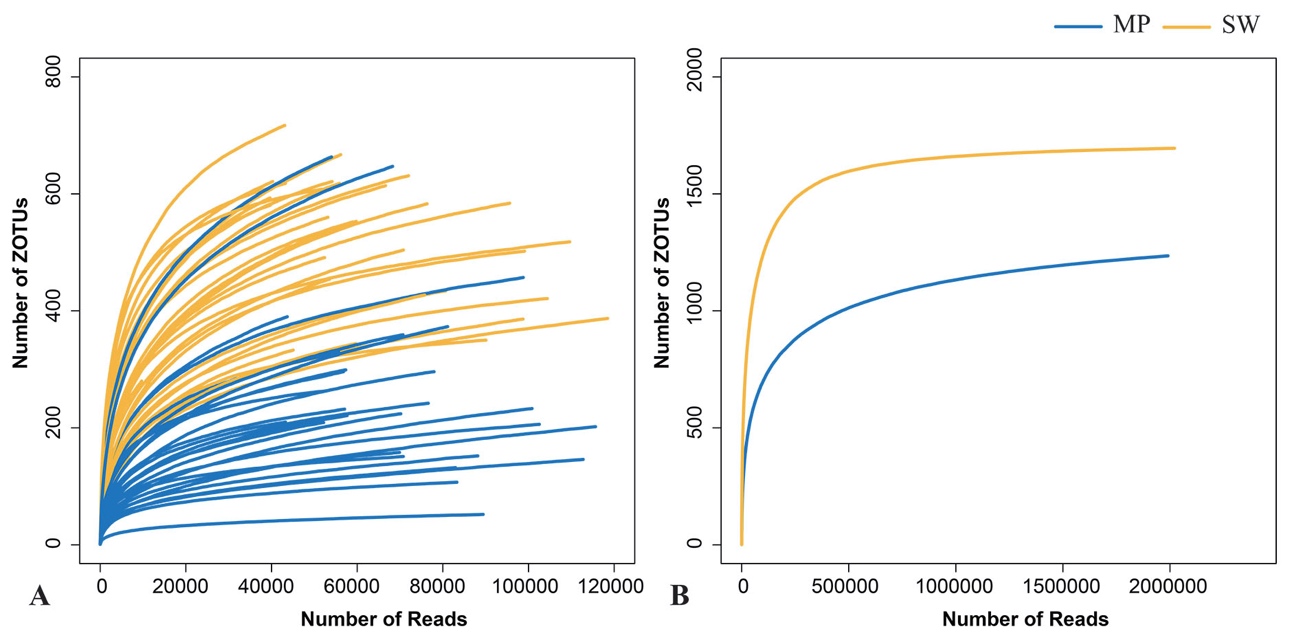


**FIGURE S2.** Alpha-diversity estimates (Shannon and Chao1) for the size-fractionated (**A, B**) samples of the melt ponds (MPs) and sea water (SW) and for the pooled (**C, D**) MP and SW samples, respectively. The line in each box plot indicates the median, the box delimits the 25th and 75th percentile. Bars in (**C**) and (**D**) without shared letters indicate significant differences at the level of p = 0.05.


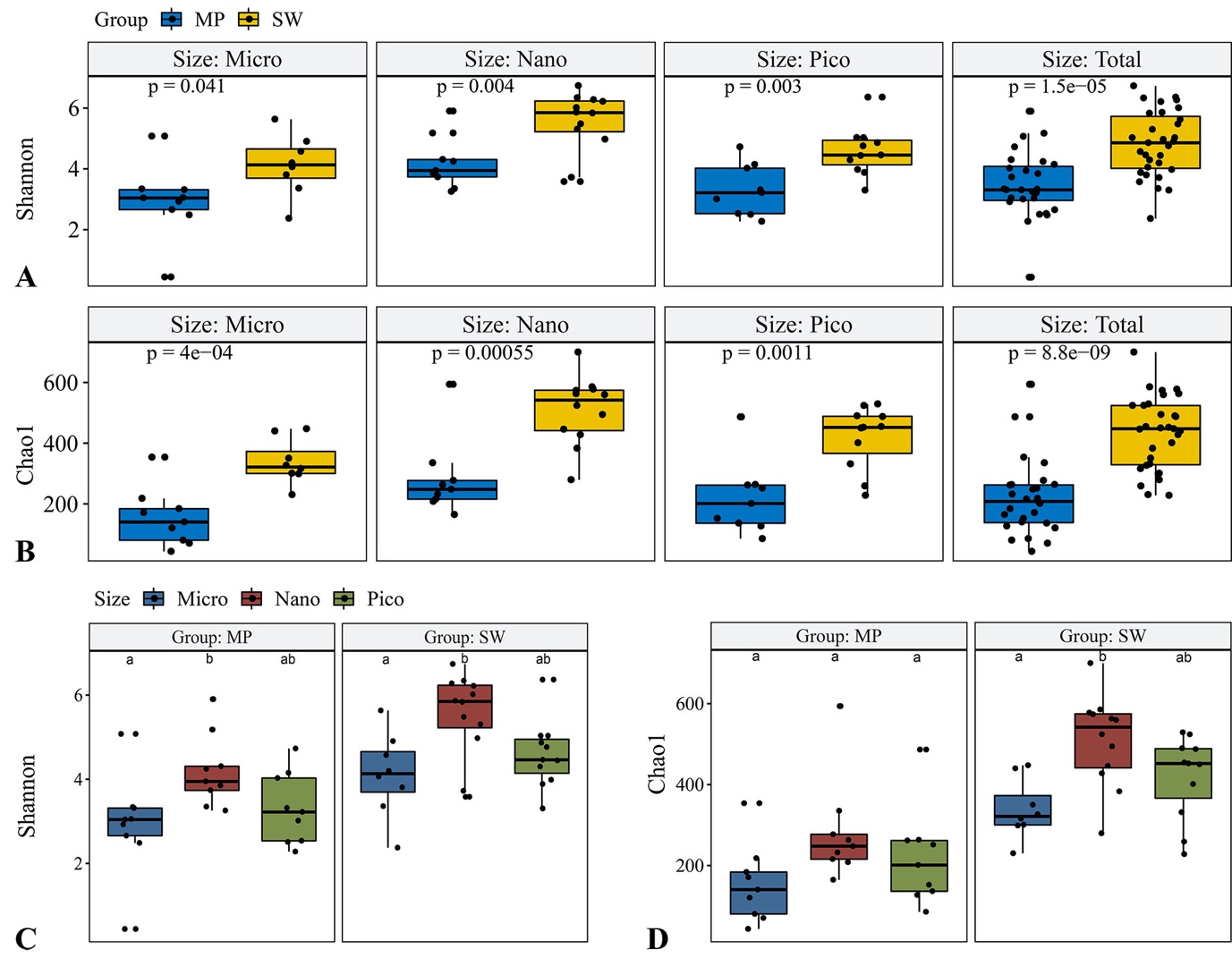


**FIGURE S3.** Relative numbers of sequences and ZOTUs of individual sea water (SW) and melt ponds (MPs) microbial eukaryotes.


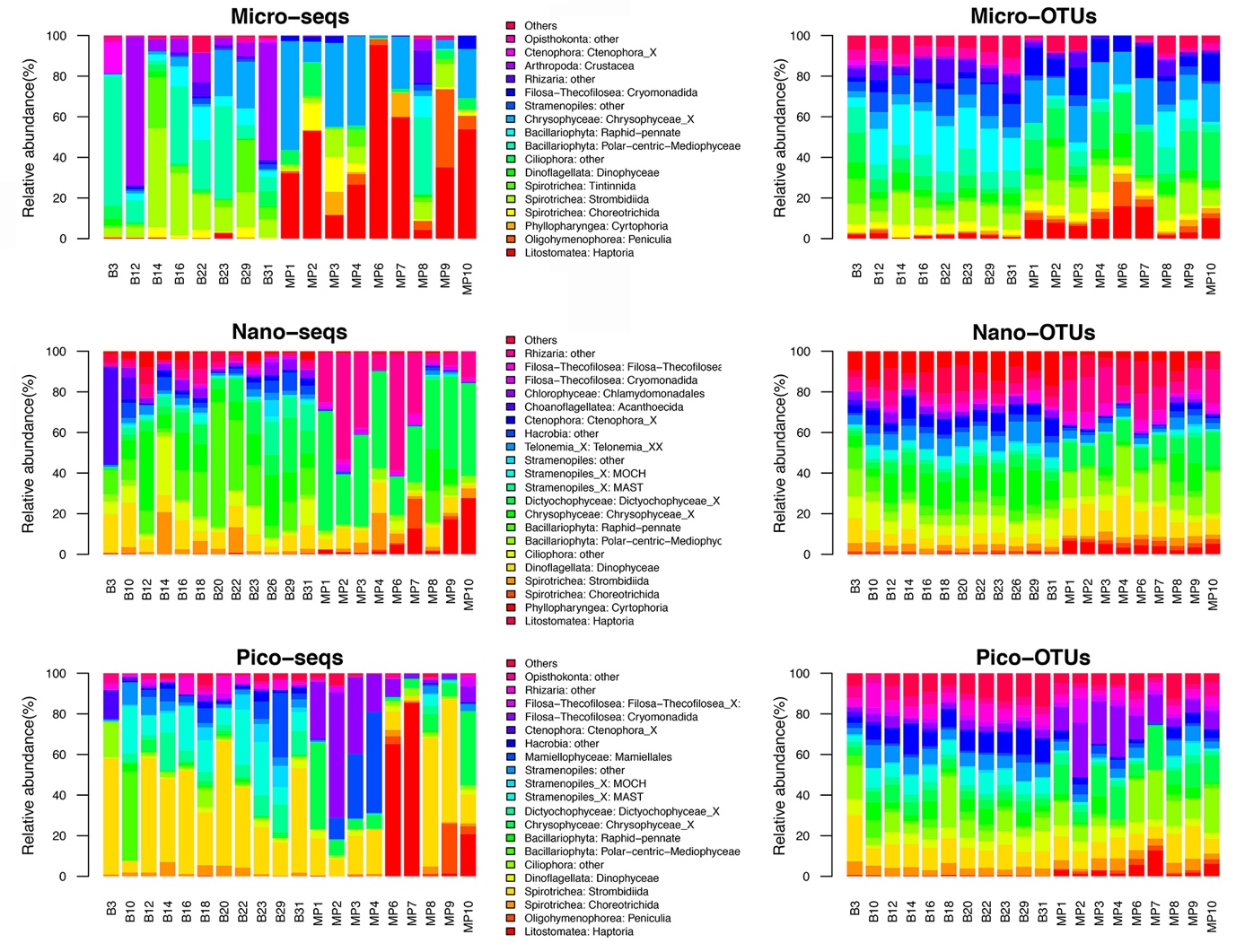


**FIGURE S4.** Venn diagram showing the shared ZOTUs among sea water (SW) and melt pond (MP) groups (**A**), among sea water (SW), open (OMP) and closed (CMP) melt ponds (**C**) and the shared ZOTUs affiliated with Ciliophora among micro-, nano-, and pico-sized subcommunities (**B**) respectively.


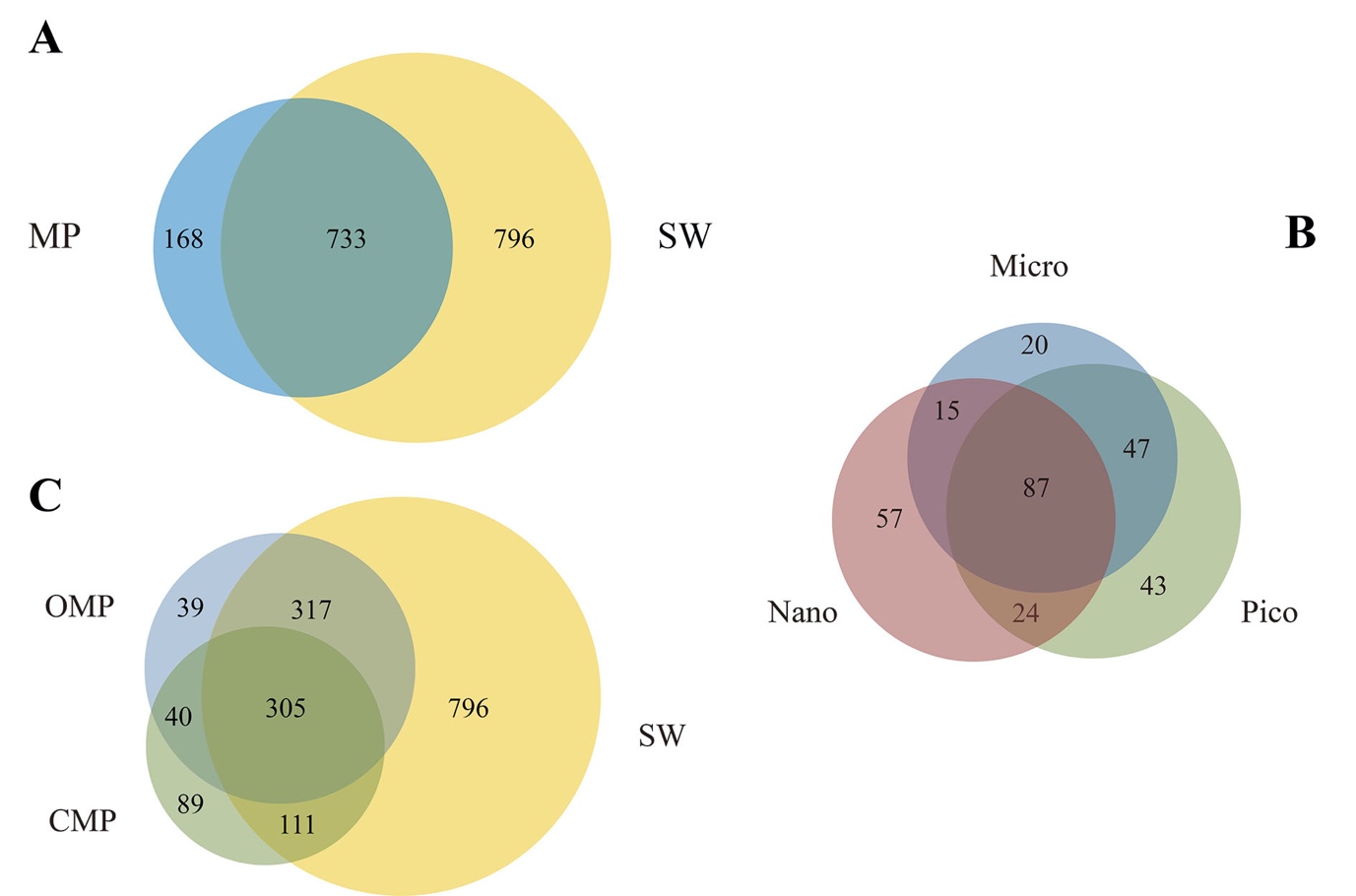


**FIGURE S5.** Community dissimilarity of sea water (SW) and melt pond (MP) samples as revealed by Bray Curtis dissimilarities.


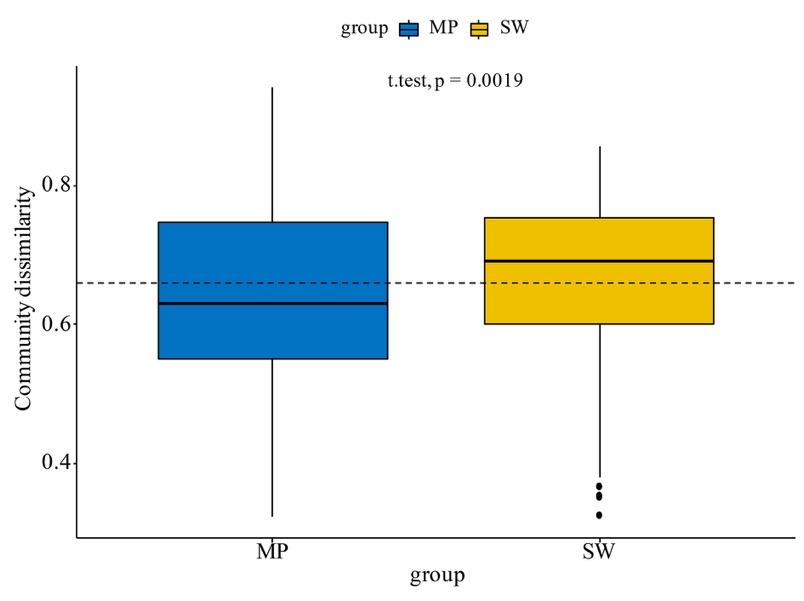


**Table S1.** Coordinates of the sampling sites and diversity parameters of the samples. *Reads after quality control; **Standardized numbers based on subsampling of 9,674 sequences without replacement.

| **Station** | **Lat.** | **Long.** | **Size** | **Sample ID** | **Reads**  **affiliated**  **with**  **microbial eukaryotes** | **ZOTUs**** | **Chao1** | **Shannon** | | **PD** | |
| --- | --- | --- | --- | --- | --- | --- | --- | --- | --- | --- | --- |
| B3 | 67.67 | -168.96 | micro | ARA07B3micro | 90124 | 197 | 299 | 3.36 | 25.95 | |  |
|  |  |  | nano | ARA07B3nano | 45022 | 197 | 280 | 3.72 | 23.03 | |  |
|  |  |  | pico | ARA07B3pico | 59948 | 163 | 228 | 3.30 | 19.54 | |  |
| B10 | 70.5 | -168.67 | nano | ARA07B10nano | 9647 | 269 | 428 | 5.48 | 29.99 | |  |
|  |  |  | pico | ARA07B10pico | 75746 | 189 | 259 | 3.89 | 21.13 | |  |
| B12 | 72.36 | -168.67 | micro | ARA07B12micro | 99177 | 257 | 350 | 2.37 | 33.11 | |  |
|  |  |  | nano | ARA07B12nano | 54139 | 398 | 574 | 5.86 | 38.53 | |  |
|  |  |  | pico | ARA07B12pico | 55996 | 358 | 524 | 5.03 | 32.08 | |  |
| B14 | 74.80 | -167.90 | micro | ARA07B14micro | 80538 | 221 | 301 | 4.20 | 25.15 | |  |
|  |  |  | nano | ARA07B14nano | 56125 | 400 | 585 | 5.84 | 34.95 | |  |
|  |  |  | pico | ARA07B14pico | 70637 | 265 | 452 | 4.76 | 26.98 | |  |
| B16 | 75.15 | -176.00 | micro | ARA07B16micro | 95656 | 310 | 448 | 4.91 | 33.33 | |  |
|  |  |  | nano | ARA07B16nano | 43089 | 493 | 700 | 6.74 | 44.48 | |  |
|  |  |  | pico | ARA07B16pico | 76281 | 292 | 401 | 3.99 | 32.43 | |  |
| B18 | 75.77 | 177.07 | nano | ARA07B18nano | 43244 | 472 | 578 | 6.28 | 44.69 | |  |
|  |  |  | pico | ARA07B18pico | 53923 | 439 | 487 | 6.37 | 36.80 | |  |
| B20 | 77.00 | 176.57 | nano | ARA07B20nano | 43864 | 306 | 446 | 3.58 | 32.58 | |  |
|  |  |  | pico | ARA07B20pico | 59084 | 306 | 450 | 4.44 | 29.54 | |  |
| B22 | 78.52 | 178.85 | micro | ARA07B22micro | 109627 | 303 | 440 | 5.63 | 34.51 | |  |
|  |  |  | nano | ARA07B22nano | 39610 | 367 | 560 | 4.97 | 37.71 | |  |
|  |  |  | pico | ARA07B22pico | 59763 | 307 | 455 | 4.46 | 31.95 | |  |
| B23 | 77.87 | -175.91 | micro | ARA07B23micro | 98728 | 189 | 316 | 4.57 | 24.01 | |  |
|  |  |  | nano | ARA07B23nano | 39777 | 411 | 563 | 6.02 | 40.83 | |  |
|  |  |  | pico | ARA07B23pico | 66800 | 356 | 529 | 4.87 | 31.20 | |  |
| B26 | 76.00 | -170.50 | nano | ARA07B26nano | 53161 | 375 | 494 | 6.22 | 32.73 | |  |
| B29 | 77.47 | -164.12 | micro | ARA07B29micro | 118451 | 179 | 230 | 4.06 | 25.21 | |  |
|  |  |  | nano | ARA07B29nano | 52387 | 300 | 383 | 5.31 | 28.51 | |  |
|  |  |  | pico | ARA07B29pico | 53569 | 252 | 332 | 4.30 | 26.56 | |  |
| B31 | 75.69 | -166.64 | micro | ARA07B31micro | 104398 | 228 | 327 | 3.81 | 31.06 | |  |
|  |  |  | nano | ARA07B31nano | 40266 | 409 | 524 | 6.34 | 37.22 | |  |
|  |  |  | pico | ARA07B31pico | 72114 | 356 | 490 | 5.03 | 34.30 | |  |
| BIC(MP10) |  |  | micro | ARA07MP10micro | 88157 | 80 | 121 | 2.66 | 9.74 | |  |
|  |  |  | nano | ARA07MP10nano | 52167 | 382 | 594 | 5.90 | 33.83 | |  |
|  |  |  | pico | ARA07MP10pico | 70740 | 178 | 262 | 4.03 | 22.35 | |  |
| BIC(MP1) | 77.99 | -176.99 | micro | ARA07MP1micro | 115659 | 86 | 171 | 3.04 | 9.87 | |  |
|  |  |  | nano | ARA07MP1nano | 57069 | 151 | 277 | 4.25 | 14.70 | |  |
|  |  |  | pico | ARA07MP1pico | 57448 | 163 | 264 | 4.15 | 17.43 | |  |
| BIC(MP2) |  |  | micro | ARA07MP2micro | 76692 | 115 | 184 | 2.93 | 14.00 | |  |
|  |  |  | nano | ARA07MP2nano | 43269 | 133 | 232 | 3.74 | 13.71 | |  |
|  |  |  | pico | ARA07MP2pico | 69912 | 82 | 127 | 3.32 | 9.41 | |  |
| BIC(MP3) |  |  | micro | ARA07MP3micro | 100827 | 95 | 218 | 3.34 | 13.53 | |  |
|  |  |  | nano | ARA07MP3nano | 57643 | 116 | 165 | 3.35 | 13.27 | |  |
|  |  |  | pico | ARA07MP3pico | 70174 | 101 | 152 | 3.01 | 10.29 | |  |
| BIC(MP4) |  |  | micro | ARA07MP4micro | 112754 | 61 | 80 | 3.05 | 6.95 | |  |
|  |  |  | nano | ARA07MP4nano | 56806 | 177 | 208 | 3.85 | 18.07 | |  |
|  |  |  | pico | ARA07MP4pico | 82878 | 68 | 86 | 2.28 | 7.82 | |  |
| BIC(MP6) |  |  | micro | ARA07MP6micro | 89399 | 25 | 43 | 0.44 | 4.87 | |  |
|  |  |  | nano | ARA07MP6nano | 52769 | 175 | 216 | 3.95 | 18.38 | |  |
|  |  |  | pico | ARA07MP6pico | 78029 | 139 | 201 | 2.51 | 18.53 | |  |
| BIC(MP7) |  |  | micro | ARA07MP7micro | 83288 | 57 | 70 | 2.48 | 6.85 | |  |
|  |  |  | nano | ARA07MP7nano | 55703 | 196 | 263 | 4.31 | 19.61 | |  |
|  |  |  | pico | ARA07MP7pico | 70763 | 86 | 137 | 2.54 | 8.87 | |  |
| BIC(MP8) |  |  | micro | ARA07MP8micro | 98777 | 235 | 354 | 5.08 | 28.01 | |  |
|  |  |  | nano | ARA07MP8nano | 53986 | 133 | 248 | 3.26 | 13.71 | |  |
|  |  |  | pico | ARA07MP8pico | 68385 | 345 | 486 | 4.73 | 34.81 | |  |
| BIC(MP9) |  |  | micro | ARA07MP9micro | 102493 | 98 | 141 | 3.31 | 10.39 | |  |
|  |  |  | nano | ARA07MP9nano | 43663 | 234 | 335 | 5.18 | 21.48 | |  |
|  |  |  | pico | ARA07MP9pico | 81080 | 162 | 251 | 3.22 | 18.38 | |  |

**Table S2.** Analysis of similarities (ANOSIM) tests of the groupings of microbial eukaryote subcommunities according to size calculated from Bray Curtis dissimilarities.

|  |  | ANOSIM | |
| --- | --- | --- | --- |
|  |  | R | P |
| Melt pond | micro vs. nano | 0.417 | 0.001 |
|  | micro vs. pico | 0.345 | 0.001 |
|  | nano vs. pico | 0.236 | 0.003 |
| Sea water | micro vs. nano | 0.460 | 0.001 |
|  | micro vs. pico | 0.798 | 0.001 |
|  | nano vs. pico | 0.484 | 0.001 |
